# Anxiety, home blood pressure monitoring, and cardiovascular events among older hypertension patients during the COVID-19 pandemic

**DOI:** 10.1038/s41440-022-00852-0

**Published:** 2022-01-21

**Authors:** Shuyuan Zhang, Yixuan Zhong, Lixin Wang, Xinhua Yin, Yufeng Li, Yunlan Liu, Qiuyan Dai, Anli Tong, Dongfeng Li, Liangqing Zhang, Ping Li, Guohui Zhang, Rongjie Huang, Jinguang Liu, Luosha Zhao, Jing Yu, Xinjun Zhang, Li Yang, Jun Cai, Weili Zhang, On behalf of the STEP Study Group

**Affiliations:** 1 State Key Laboratory of Cardiovascular Disease, Hypertension Center, FuWai Hospital, National Center for Cardiovascular Diseases, Peking Union Medical College & Chinese Academy of Medical Sciences, Beilishi Road 167, Xicheng District, Beijing, 100037 China; 2 The First Affiliated Hospital of Hebei North University, Hebei, 075061 China; 3 Southern University of Science and Technology Hospital, Shenzhen, 518000 China; 4 First Affiliated Hospital of Harbin Medical University, Heilongjiang, 150081 China; 5 Beijing Pinggu Hospital, Beijing, 101200 China; 6 The First Hospital of Kunming, Yunnan, 650011 China; 7 Shanghai General Hospital, Shanghai Jiaotong University, Shanghai, 200080 China; 8 Peking Union Medical College Hospital, Chinese Academy of Medical Sciences & Peking Union Medical College, Beijing, 100730 China; 9 Wuxiang County People’s Hospital, Shanxi, 046300 China; 10 Shanxi Cardiovascular Hospital, Shanxi, 030000 China; 11 2nd Affiliated Hospital of Nan Chang University, Jiangxi, 330006 China; 12 Zhenjiang First People’s Hospital, Jiangsu, 212002 China; 13 The First Affiliated Hospital of Guangxi Medical University, Guangxi, 530021 China; 14 Huizhou Municipal Central Hospital, Guangdong, 516001 China; 15 First Affiliated Hospital of Zhengzhou University, Henan, 450000 China; 16 Lanzhou University Second Hospital, Gansu, 730030 China; 17 West China Hospital, Sichuan University, Sichuan, 610041 China; 18 Yan’an Hospital affiliated Kunming Medical University, Yunnan, 650101 China

**Keywords:** Coronavirus disease 2019 (COVID-19), Anxiety, Home blood pressure, Cardiovascular disease, Smartphone-based application

## Abstract

The global coronavirus disease 2019 (COVID-19) pandemic has led to a health crisis. It remains unclear how anxiety affects blood pressure (BP) and cardiovascular risk among older patients with hypertension. In this study, we extracted longitudinal data on home BP monitored *via* a smartphone-based application in 3724 elderly patients with hypertension from a clinical trial (60–80 years; 240 in Wuhan and 3484 in non-Wuhan areas) to examine changes in morning BP during the COVID-19 outbreak in China. Anxiety was evaluated using Generalized Anxiety Disorder-7 item scores. Changes in morning systolic BP (SBP) were analyzed for five 30-day periods during the pandemic (October 21, 2019 to March 21, 2020), including the pre-epidemic, incubation, developing, outbreak, and plateau periods. Data on cardiovascular events were prospectively collected for one year. A total of 262 individuals (7.0%) reported an increased level of anxiety, and 3462 individuals (93.0%) did not. Patients with anxiety showed higher morning SBP than patients without anxiety, and the between-group differences in SBP change were +1.2 mmHg and +1.7 mmHg during the outbreak and plateau periods (*P* < 0.05), respectively. The seasonal BP variation in winter among patients with anxiety was suppressed during the pandemic. Anxious patients had higher rates of uncontrolled BP. During the 1-year follow-up period, patients with anxiety had an increased risk of cardiovascular events with a hazard ratio of 2.47 (95% confidence interval, 1.10–5.58; *P* = 0.03). In summary, COVID-19-related anxiety was associated with a short-term increase in morning SBP among older patients and led to a greater risk of cardiovascular events. (ClinicalTrials. gov number, NCT03015311).

## Introduction

Hypertension affects more than 50% of older people aged ≥60 years in China and worldwide [1, 2]. Older hypertensive patients are more likely to have anxiety, and the comorbidity of mental conditions and hypertension is associated with higher cardiovascular mortality than hypertension alone [3]. Effective blood pressure (BP) control in older patients can reduce the risk of cardiovascular disease (CVD) and mortality. BP varies both in the short term (e.g., days) and long term (e.g., months, quarters, or years). Therefore, home BP monitoring plays an important role in hypertension management and has been strongly recommended in recent hypertension guidelines as an adjunct to office BP measurement [4, 5].

The coronavirus disease 2019 (COVID-19) pandemic has led to more than 2 million deaths worldwide [6]. Older hypertensive patients are more vulnerable to severe COVID-19 outcomes [7, 8] and may experience large fluctuations in BP along with psychological stress such as anxiety, depression, or other negative emotions [9]. Effective management of BP during the COVID-19 pandemic is very important; however, there is a lack of evidence from large-scale, prospective studies about whether COVID-19-related anxiety affects BP control and cardiovascular risk.

In China, the first COVID-19 case was detected and reported in Wuhan in early December 2019. The outbreak started in early January 2020 and then rapidly spread nationally, reaching a peak of infection in February 2020. Owing to the actions taken by the Chinese government, such as implementing traffic restrictions and building FangCang shelter hospitals, the pandemic came under control in late March 2020 [10]. In this study, we used data from a randomized clinical trial of BP management leveraging a smartphone-based application (app) to examine whether anxiety was associated with large fluctuations in home BP among older patients with hypertension before and during the COVID-19 outbreak. We further investigated cardiovascular outcomes after the pandemic was controlled between patients with anxiety and without anxiety in Wuhan and other provinces of China.

## Methods

### Categorization of COVID-19 pandemic period

In this study, the pandemic timeline was categorized into five 30-day periods on the basis of the dynamics of COVID-19 and the actions taken by the Chinese government [10, 11], including the pre-epidemic period (October 21 to November 20 2019, as the reference), incubation period (November 21 to December 20, 2019), developing period (December 21, 2019 to January 20, 2020), outbreak period (January 21 to February 20, 2020), and plateau period (February 21 to March 21, 2020) (Supplementary Fig. 1).

### Study participants

The participants were from the Strategy of Blood Pressure Intervention in Elderly Hypertensive Patients (STEP) study, a multicenter clinical trial of BP management in elderly patients with hypertension in China (ClinicalTrials.gov. NCT03015311) [12]. Briefly, a total of 8511 patients with essential hypertension aged 60–80 years were enrolled from January 10 to December 31, 2017, at 42 collaborating clinical centers throughout China and were followed up every 3 months until the end of the study (December 31, 2020). The inclusion and exclusion criteria are described in the Supplementary Methods. A geographic map of clinical centers is shown in Supplementary Figure 2. This study was approved by the Ethics Committee of FuWai Hospital, and all participants provided written informed consent.

All patients were provided with the same validated Omron automatic BP monitor (HEM-9200T, Omron Healthcare, Kyoto, Japan) that meets the requirement of the European Society of Hypertension International Protocol revision 2010 (ESH-IP revision 2010) [13]. This monitor has a bluetooth function to allow patients to upload home BP readings to a data center via a smartphone-based app (http://www.gaoxinhealth.com/). In the present study, longitudinal data of home morning BP were extracted to examine how the recent COVID-19 outbreak affected BP control. An online survey on the mental health status of patients in Wuhan and other provinces of China was conducted in March 2020 during the COVID-19 outbreak.

**Fig. 1:**
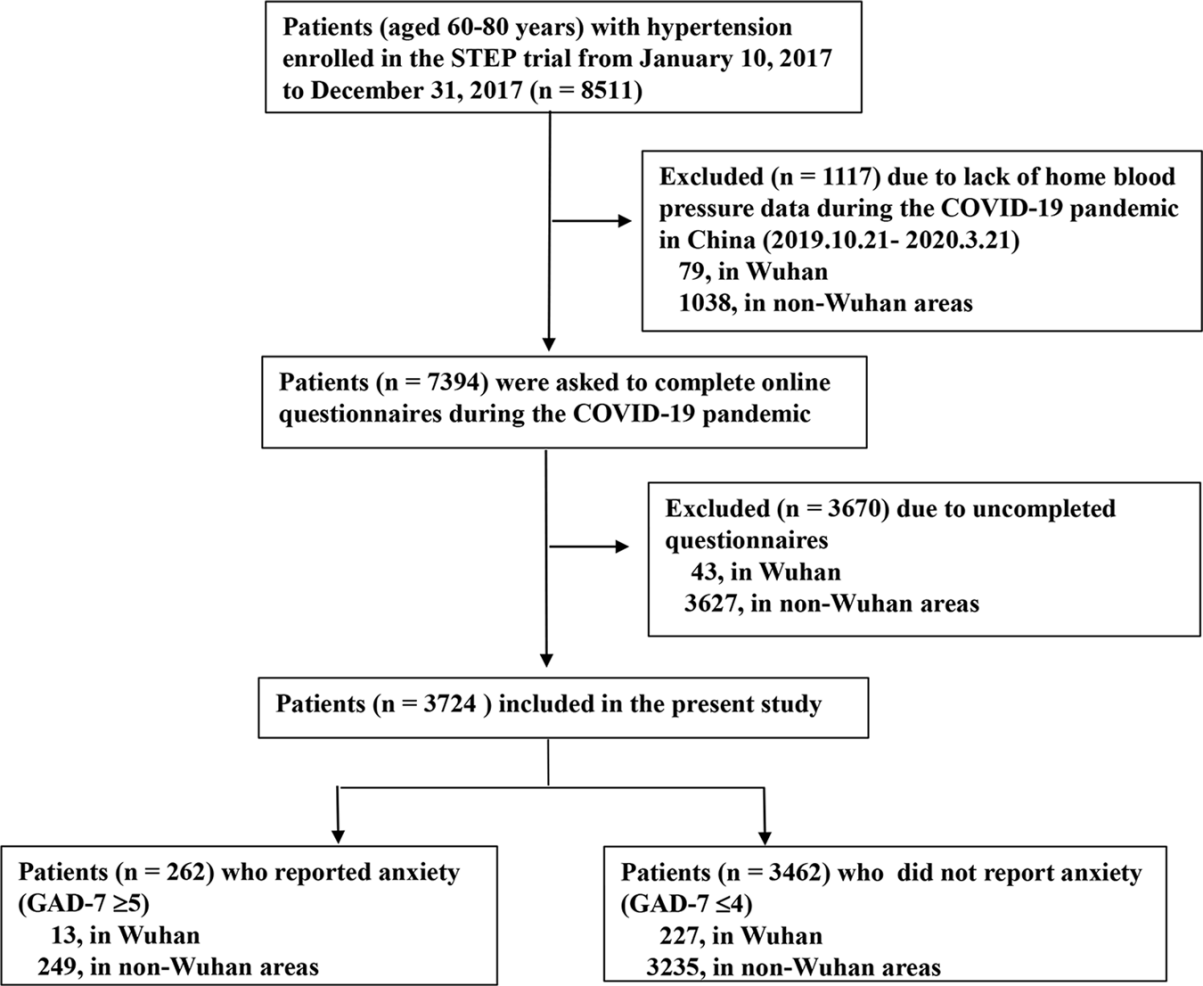
Flowchart of the present study. Abbreviations: STEP the strategy of blood pressure intervention in older hypertensive patients, COVID-19 coronavirus disease 2019, BP blood pressure

A total of 1117 patients were excluded due to a lack of BP measurement data during the pandemic, and 3670 patients were excluded due to incomplete online survey responses. Thus, 3724 patients were included in the analysis of BP changes (*n* = 240 in Wuhan and *n* = 3484 in non-Wuhan areas in China; Fig. 1). The included patients had at least one BP measurement at each period from the pre-epidemic to the plateau period (October 21, 2019 to March 21, 2020). There were modest differences in several clinical features between the included and excluded patients; for example, the excluded patients were older (69.0 *versus* 68.3 years), were less educated (middle school or below: 59.9% *versus* 52.9%), and had a higher prevalence of coronary heart disease history (7.1% *versus* 5.3%) than the included patients. There were no significant differences in average morning systolic BP (SBP) and diastolic BP (DBP) levels in the pre-epidemic period between the included and excluded patients (Supplementary Table 1).

### Anxiety survey during the COVID-19 pandemic

The mental health status of patients during the COVID-19 pandemic was assessed using the 7-item Generalized Anxiety Disorder Scale (GAD-7) (Supplementary Methods). The GAD-7 is a brief self-report scale that has demonstrated good reliability for the general population [14] and has also been validated for the older population [15]. Participants were asked to answer how frequently they had been bothered by various symptoms over the previous 2 weeks. Response options were ‘not at all’, ‘several days’, ‘more than half the days’, and ‘nearly every day’, scored as 0, 1, 2, and 3, respectively. The summary GAD-7 score ranges from 0 to 21. In this study, respondents who scored GAD-7≥5 were identified as having moderate or severe anxiety, and respondents who scored GAD-7 ≤ 4 were considered to not have anxiety [15].

The survey also investigated the change in physical activity (≥7 times, 5–7 times, 2–4 times, or once a week) before and during the COVID-19 period. The app platform carried out remote education lectures by physicians on cardiovascular health in the pandemic situation, for example, how to maintain a healthy lifestyle at home, how to alleviate a tense mood, and what effects COVID-19 has on the cardiovascular system. In the survey, we also investigated whether patients attended these online lectures via the app platform.

### Home BP measurements and variability

The app platform used in this study was designed to automatically sync with the home BP of patients and to send WeChat reminders to patients if BP was not measured regularly and transmitted to the data center. The app had a patient portal and a doctor portal. Both doctors and patients can track daily home BP readings and graphical trends in BP change via a predesigned module in the app during the follow-up period. For BP measurement, patients were required to rest for at least 5 min in a seated position before BP was measured three times (at least 1 min apart). All patients were recommended to measure their home BP for at least 1 day per week. Home BP in the morning was measured within 1 h of waking, usually from 6:00 a.m. to 10:00 a.m., after urination but before antihypertensive drug intake and breakfast. Because China spans five time zones and older people usually get up early, for the present analysis, we collected BP data in the morning from 5:00 a.m. to 10:00 a.m.

During each phase of the COVID-19 pandemic, intraindividual reading-to-reading BP variability was evaluated using the coefficient of variation of BP (expressed as standard deviation [SD]/mean × 100%). The difference (maximum–minimum) in morning BP was also assessed as an indicator of BP variability, which might have associations with cardiovascular outcome [16].

### Assessment of endpoints

The primary cardiovascular outcome of the STEP trial was a composite of acute coronary syndrome (acute myocardial infarction and hospitalization for unstable angina), stroke (ischemic or hemorrhagic), acute decompensated heart failure, coronary revascularization, atrial fibrillation, or death from cardiovascular causes. The definitions and evaluation criteria of the study endpoints are outlined in the Supplementary Methods. In the present study, we collected primary outcomes that occurred from the COVID-19 incubation period to the end of the trial (November 20, 2019 to December 31, 2020).

### Assessment of covariates

At baseline, all participants completed a standardized questionnaire, and clinical characteristics were assessed, including age, sex, weight, height, waist circumference, educational level, smoking status, alcohol intake, medical history, and current medication treatment. Data on the use of antihypertensive agents (i.e., calcium channel blockers, angiotensin receptor blockers, beta-blockers, and hydrochlorothiazide), hypoglycemic agents, statins, and aspirin were collected during the follow-up period. Regimen change was considered if a patient increased or decreased the dose of antihypertensive agent, received an additional agent, or switched to a different agent class. The antihypertensive treatments are detailed in the Supplementary Methods.

### Statistical analysis

Clinical characteristics of participants were compared between groups using chi-square tests for categorical variables (expressed as numbers [percentages]) and *t* tests for quantitative variables (expressed as the mean ± SD). Patients were categorized into two groups according to GAD-7 scale scores: without anxiety (GAD-7 score ≤4) and with anxiety (GAD-7 score ≥5). In this study, considering the mean difference in average morning SBP change (∆SBP) in the patients with anxiety (mean, 0.9 mmHg; SD, 6.9) and in the patients without anxiety (mean, −0.1 mmHg; SD, 5.7) during the COVID-19 pandemic, a sample size of 3724 participants would provide >80% power, with a two-sided alpha level of 0.05 to detect a between-group difference of >1 mmHg in SBP change. The power analysis was conducted by the PASS software program (www.ncss.com).

Morning SBP data are presented as the mean (95% confidence interval [CI]). Linear mixed models (adjusted for age, sex, and body mass index [BMI]) were used to compare morning SBP at the individual level at each period of the pandemic between patients who reported an increased level of anxiety and those who did not, with random intercepts to account for repeated measurements within the same patient. Time (five periods of the pandemic) and mental status group (with or without anxiety) were fitted as fixed effects, and an interaction term between time and mental status group was included in the model. To compare the between-group difference in the change in average morning SBP (ΔSBP) between patients with anxiety and without anxiety at the various time points of the pandemic, a linear regression model was used with adjustment for age, sex, and BMI. Subgroup analysis stratified by Wuhan or non-Wuhan area was also conducted. SBP variability within patients, evaluated by the coefficient of variation of SBP and the difference in SBP, was compared between the two groups using Student’s *t* test.

The proportions of patients according to categories of the number of drugs and drug classes were compared using the chi-square test. The number of antihypertensive drugs was compared over the five time periods of the pandemic between the two groups using a generalized linear model adjusted for age, sex, and BMI.

Hazard ratios with 95% CIs were calculated using a multivariate Cox proportional hazard regression model to evaluate the association between anxiety and the risk of cardiovascular events among older patients with hypertension after the occurrence of the pandemic. We analyzed the clinical characteristics of patients to assess the potential variables that might influence the relationship between anxiety and cardiovascular outcome. The analytical models were first adjusted for age, sex, BMI, education level, and baseline SBP during the pre-epidemic period and then further adjusted for the usage of lipid-lowering agents, hypoglycemic agents, aspirin, and changes in average morning SBP. The person-year of follow-up for each patient was determined from the date of cardiovascular events, death, loss to follow-up, or the last follow-up, whichever came first.

A two-tailed *P* value of <0.05 was considered significant. Analyses were performed using the Statistical Package for the Social Sciences (version 20.0; IBM Corp., NY, USA).

## Results

### Clinical characteristics of studied patients

This study included a total of 3724 patients with hypertension aged 60–80 years (240 in Wuhan and 3484 in other provinces of China); of these, 262 (7.0%) reported an increased level of anxiety, and 3462 (93.0%) did not. The mean age of patients was 68.3 (SD 4.7) years. A total of 28.2% of patients were aged ≥70 years, 46.5% were men, 19.5% had prior diabetes mellitus, 5.3% had prior coronary heart disease, and 63.6% were at high risk of coronary heart disease (10-year Framingham risk score ≥15%). There were no significant differences in clinical characteristics between patients without anxiety (GAD-7 ≤ 4) and with anxiety (GAD-7 ≥ 5) during the pre-epidemic period (Table 1). Additional stratification analysis by Wuhan or non-Wuhan areas showed that in Wuhan, patients with anxiety had a higher education level (Supplementary Table 2).

**Table 1:** Baseline characteristics of patients with anxiety and without anxiety in the present study*

Characteristics in the pre-epidemic phase	Without anxiety (*n* = 3462)	With anxiety (*n* = 262)	*P* Value^†^
Age, years	68.3 ± 4.7	68.7 ± 4.7	0.19
Distribution of age, No. (%)			
60–70 years	2492 (72.0)	182 (69.5)	0.38
≥70 years	970 (28.0)	80 (30.5)	
Men, No. (%)	1611 (46.5)	120 (45.8)	0.82
Body mass index, kg/m^2^	25.7 ± 3.2	25.7 ± 3.2	0.80
Morning SBP, mmHg	130.9 ± 9.3	131.8 ± 10.0	0.15
<140 mmHg, No. (%)	2864 (82.7)	204 (77.9)	0.13
140–149 mmHg, No. (%)	534 (15.4)	51 (19.4)	
>150 mmHg, No. (%)	64 (1.8)	7 (2.7)	
Morning DBP, mmHg	79.8 ± 7.6	79.9 ± 8.0	0.78
Fasting glucose, mmol/L	6.1 ± 1.6	6.3 ± 2.0	0.19
Lipids profile, mmol/L			
Total cholesterol	4.9 ± 1.1	5.0 ± 1.2	0.37
Triglycerides	1.2 (0.8–1.8)	1.6 (0.8–1.8)	0.51
HDL-C	1.3 ± 0.3	1.3 ± 0.3	0.71
LDL-C	2.7 ± 0.9	2.7 ± 1.0	0.83
Educational level, No. (%)			
Middle school or below	1834 (53.0)	136 (51.9)	0.74
High school or above	1628 (47.0)	126 (48.1)	
Smoking status, No. (%)			
Never	2466 (71.4)	198 (75.6)	0.08
Former	423 (12.2)	35 (13.3)	
Current	565 (16.4)	29 (11.0)	
Alcohol intake, No. (%)			
Never	2337 (67.7)	182 (69.5)	0.76
Former	174 (5.0)	11 (4.2)	
Current	943 (27.3)	69 (26.3)	
Medical history, No. (%)			
Diabetic mellitus	674 (19.5)	54 (20.6)	0.65
Coronary heart disease	184 (5.3)	14 (5.3)	0.98
Medication usage, No. (%)			
Lipid-lowering agents	866 (25.0)	65 (24.8)	0.94
Hypoglycemic agents	630 (18.2)	48 (18.3)	0.96
Aspirin	327 (9.4)	21 (9.4)	0.44
The 10-year risk of CVD^§^, %	19.2 ± 8.4	19.2 ± 8.7	0.70
The 10-year risk of CVD ≥ 15%, No. (%)^§^	2199 (63.9)	169 (64.8)	0.78

Values were given as mean ± SD, number (%), or median (interquartile range)

*SBP* systolic blood pressure, *DBP* diastolic blood pressure, *HDL-C* high-density lipoprotein cholesterol, *LDL-C* low-density lipoprotein cholesterol, *CVD* cardiovascular disease

*Patients were classified into two groups according to the generalized anxiety disorder scale-7 scores, which of ≤4 and ≥5 were interpreted as representing patients without or with anxiety, respectively.

^†^*P* values were calculated by Student *t* test or Mann–Whitney nonparametric test for quantitative variables, or by Chi-square test for qualitative variables, when appropriate

^§^The 10-year CVD risk was estimated by Framingham risk score, and patients with a ≥15% risk score were considered at high risk

### BP fluctuations in relation to anxiety during the pandemic

Patients who reported anxiety paid more attention to self-monitoring of BP during the pandemic. The average number of home BP measurements and transmissions increased rapidly to 4.3–4.7 days/week per patient after the outbreak on January 21, 2020. In contrast, for patients who did not report anxiety, the frequency of BP measurements remained stable during the pandemic, at 4.2–4.5 days/week per patient (Supplementary Fig. 3).

**Fig. 2:**
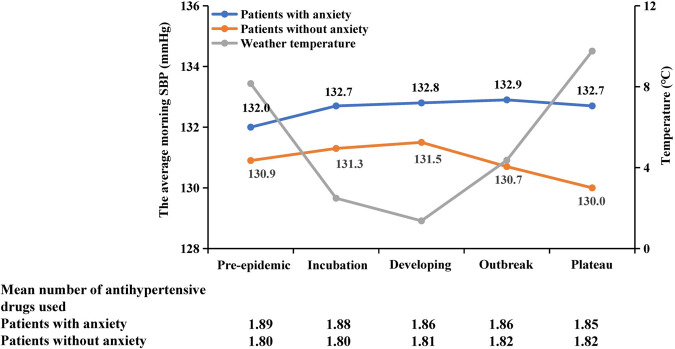
Trajectory pattern of morning SBP and antihypertensive medication in older patients with anxiety compared with those without anxiety during the pandemic. Abbreviations: SBP systolic blood pressure. The timeline of COVID-19 in China was classified as the pre-epidemic period as the reference (October 21 to November 20, 2019), incubation period (November 21 to December 20, 2019), developing period (December 21, 2019 to January 20, 2020), outbreak period (January 21 to February 20, 2020), and plateau period (February 21 to March 21, 2020). The values in the graphs indicate the adjusted mean monthly average morning SBP within each period of the pandemic among patients with anxiety (blue line) and without anxiety (orange line) after adjustment for age, sex, and body mass index. The gray line indicates the average temperature of the areas where the participants lived during the pandemic

There was seasonal SBP variation among patients without anxiety, whereas seasonal BP variation in patients with anxiety was suppressed during the COVID-19 pandemic. There was a significant increase in the morning SBP trend during the developing, outbreak, and plateau periods compared with the pre-epidemic period among patients with anxiety compared with patients without anxiety (Fig. 2). Changes in average morning SBP (∆SBP) were significantly greater in patients with anxiety; after adjusting for age, sex, BMI, and baseline SBP during the pre-epidemic period, the between-group differences in ∆SBP were +1.2 mmHg and +1.7 mmHg during the COVID-19 outbreak and plateau periods, respectively (*P* < 0.05) (Table 2). Further stratified analysis showed that in non-Wuhan areas of China, changes in average morning SBP were significantly higher in patients with anxiety during the COVID-19 period. However, there were no significant differences between the two groups at the five time periods of the pandemic in Wuhan (Supplementary Table 3 and Fig. 4), which could be partly explained by the small number of patients with anxiety. In addition, the morning SBP data measured in the same months in the previous year (October 21, 2018 to March 21, 2019) were extracted for sensitivity analysis to examine whether the relation between anxiety and the morning SBP trend was influenced by seasonal variation. The data showed that the average morning SBP had a seasonal BP variation and fluctuated <1 mmHg along with the change in temperature (Supplementary Fig. 5).

**Table 2:** Changes in average morning SBP of patients with anxiety and without anxiety during the COVID-19 period

The COVID-19 period	Adjusted mean (95% CI) of SBP, mmHg*			Adjusted mean difference (95% CI) of SBP (∆SBP), mmHg^‡^		The between-group difference in ∆SBP	*P*^§^
	Without anxiety (GAD-7 ≤ 4) (*n* = 3462)	With anxiety (GAD-7 ≥ 5) (*n* = 262)	P*	Without anxiety (GAD-7 ≤ 4) (*n* = 3462)	With anxiety (GAD-7 ≥ 5) (*n* = 262)		
Pre-epidemic period	130.9 (130.6, 131.2)	132.0 (130.9, 133.2)	0.07				
Incubation period	131.3 (130.9, 131.6)^†^	132.7 (131.5, 133.9)	0.03	0.4 (0.2, 0.6)	0.6 (−0.2, 1.3)	0.2 (−0.6, 1.0)	0.58
Developing period	131.5 (131.1, 131.8)^†^	132.8 (131.7, 134.0)^†^	0.05	0.5 (0.3, 0.8)	0.7 (−0.1, 1.6)	0.2 (−0.7, 1.1)	0.65
Outbreak period	130.7 (130.4, 131.0)^†^	132.9 (131.7, 134.1)^†^	<0.001	−0.3 (−0.5, −0.02)	0.9 (−0.02, 1.8)	1.2 (0.3, 2.2)	0.01
Plateau period	130.0 (129.6, 130.3)^†^	132.7 (131.6, 133.9)	<0.001	−0.9 (−1.2, −0.7)	0.8 (−0.2, 1.7)	1.7 (0.7, 2.7)	<0.001

The pandemic timeline of the COVID-19 in China was classified as the pre-epidemic period as the reference (October 21 to November 20, 2019), incubation period (November 21 to December 20, 2019), developing period (December 21, 2019 to January 20, 2020), outbreak period (January 21 to February 20, 2020), and plateau period (February 21 to March 21, 2020)

*SBP* systolic blood pressure, *COVID-19* coronavirus disease 2019, *CI* confidence interval, *GAD-7* generalized anxiety disorder scale-7

*Adjusted mean (95% CI) of SBP was calculated by linear mixed model after adjustment for age, sex, and body mass index, and *P* value was compared between patients without anxiety and patients with anxiety

^†^*P* < 0.05, each period of epidemic *versus* the pre-epidemic period (as the reference group), calculated by linear mixed model adjusting for age, sex, and body mass index.

^‡^Adjusted mean difference (95% CI) of SBP (∆SBP) was calculated as the change of average morning SBP from pre-epidemic period to each time period of COVID-19

^§^*P* value was compared between patients with anxiety and without anxiety by linear regression model after adjustment for age, sex, and body mass index

The rates of uncontrolled BP (≥140/90 mmHg) were significantly higher in patients with anxiety than in patients without anxiety: 23.3%, 21.8%, 21.8%, and 29.8% in patients with anxiety and 20.4%, 20.8%, 18.5%, and 18.5% in patients without anxiety in the incubation, developing, outbreak, and plateau periods of the COVID-19 pandemic, respectively (Supplementary Table 4). No significant differences were found in home BP variability (as evaluated by the coefficient of variability of morning SBP and the difference in morning SBP) between patients with anxiety and without anxiety at each period of the pandemic (Supplementary Table 5). In this study, the average morning DBP of patients with or without anxiety remained stable at approximately 80.3 mmHg and 79.8 mmHg, respectively, during the pandemic, whereas there was a significant between-group difference in the change in morning DBP (∆DBP) of 1 mmHg during the plateau period of COVID-19 (*P* = 0.001) (Supplementary Table 6), indicating that anxiety from the COVID-19 outbreak might have a lag effect on the change in DBP level.

Regarding BP medication, the number of antihypertensive drugs used by patients with anxiety was higher than that used by patients without anxiety, but the difference did not reach statistical significance (Fig. 2). There was no significant difference in regimen change (increased dosage or agent addition) between patients with or without anxiety (Supplementary Table 7).

### Occurrence of cardiovascular events during the pandemic

**Fig. 3:**
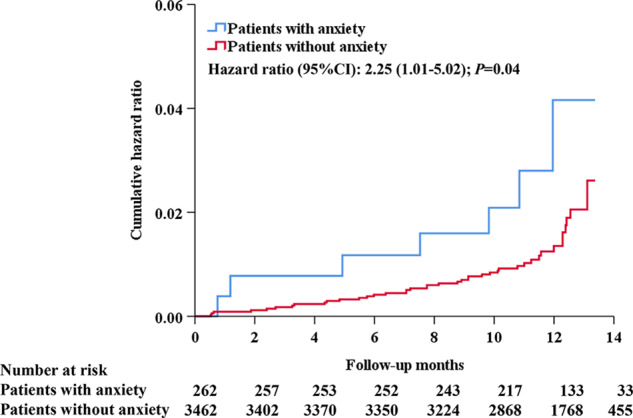
Kaplan–Meier curves of total cardiovascular events in patients with anxiety compared with patients without anxiety during the COVID-19 period. CI confidence interval, COVID-19 coronavirus disease 2019. Patients with anxiety showed a higher risk for the incidence of total cardiovascular events than patients without anxiety during the 1-year follow-up, including acute coronary syndrome (acute myocardial infarction and hospitalization for unstable angina), stroke (ischemic or hemorrhagic), acute decompensated heart failure, coronary revascularization, atrial fibrillation, or death from cardiovascular causes

During the 1-year follow-up period from the COVID-19 incubation period (November 21, 2019) to the end of the trial (December 31, 2020), a composite of cardiovascular events occurred in 44 (1.3%) patients in the nonanxiety group and 7 (2.7%) patients in the anxiety group. Anxiety status was associated with an increased risk of incident cardiovascular events during the COVID-19 pandemic (Fig. 3), with a hazard ratio of 2.47 (95% CI, 1.10–5.58; *P* = 0.03) after adjustment for age, sex, BMI, education level, baseline SBP during the pre-epidemic period, changes in average morning SBP, and the usage of lipid-lowering agents, hypoglycemic agents and aspirin (Table 3).

**Table 3:** Comparison of incidence of total cardiovascular events in patients with anxiety and without anxiety during the COVID-19 period

Outcomes	Without anxiety (GAD-7 ≤ 4)	With anxiety (GAD-7 ≥ 5)	*P* value
Patients, *n*	3462	262	
Total CVD*, *n*	44	7	
Person-years	3065	230	
HR (95% CI), model I^†^	1.0	2.32 (1.03–5.18)	0.04
HR (95% CI), model II^‡^	1.0	2.47 (1.10–5.58)	0.03

*HR* hazards ratio, *CI* confidence interval, *COVID-19* coronavirus disease 2019, *CVD* cardiovascular disease, GAD-7 generalized anxiety disorder scale-7

*Total CVD events were collected which occurred from November 21, 2019 to December 31, 2020, including a composite of acute coronary syndrome (acute myocardial infarction and hospitalization for unstable angina), stroke (ischemic or hemorrhagic), acute decompensated heart failure, coronary revascularization, atrial fibrillation, or death from cardiovascular causes

^†^HR (95% CI) and *P* value were calculated using the Cox proportional hazards regression model, and model I was adjusted for age, sex, body mass index, education level, and morning SBP during the pre-epidemic period.

^‡^Model II was further adjusted for the usage of lipid-lowering agents, hypoglycemic agents, and Aspirin, and changes in average morning SBP

### Efficiency of BP monitoring *via* the app during the pandemic

Logs of doctors and patients accessing the BP module *via* the app were analyzed to assess the efficiency of smartphone-based home BP monitoring during the pandemic. Overall, the average number of app visits per doctor fell substantially and was 114.3, 90.7, 88.8, and 106.2 times per month during the pandemic compared with 175.4 times per month during the pre-epidemic period (Supplementary Fig. 6). In contrast, the frequency of checking BP by patients via the app showed an increasing trend compared with the pre-epidemic period, and overall, it was higher in patients without anxiety than in those with anxiety (Supplementary Fig. 7).

The app platform carried out remote cardiovascular health education during the pandemic, and the online survey showed that the proportions of patients attending remote education were 60.3% in the anxiety group and 61.6% in the nonanxiety group. There was no significant difference in morning SBP during the pandemic between patients attending online education and those not attending online education (Supplementary Table 8). Changes in the frequency of physical activity during the pandemic were also surveyed. The proportions of patients with decreased physical activity during the COVID-19 outbreak were 37.8% in patients with anxiety and 41.8% in patients without anxiety. Linear regression analysis showed that changes in physical activity were not related to fluctuations in morning SBP during the pandemic in patients with anxiety or without anxiety (Supplementary Table 9).

## Discussion

In this study, we used longitudinal data of home BP monitored *via* a smartphone-based app in a total of 3724 elderly patients with hypertension from the STEP trial to evaluate the association of anxiety status with BP fluctuations and cardiovascular events during the COVID-19 outbreak in China. The time period from October 2019 to March 2020 was divided into five 30-day phases, including the pre-epidemic, incubation, developing, outbreak, and plateau periods. Our data first provided evidence that compared with patients who did not report anxiety, patients with anxiety had higher average morning SBP during the pandemic and an increased risk of incident cardiovascular events during the 1-year follow-up. The rates of uncontrolled BP in patients with anxiety were higher than those in patients without anxiety. As expected, the frequency of checking BP by patients via the app showed an increasing trend compared with that during the pre-epidemic period.

The strengths of this study include the use of a large cohort, smartphone-based home BP monitoring, comparison of multiple time periods before and during the pandemic, and further follow-up of cardiovascular outcomes.

The majority of epidemiological studies suggest a positive association between anxiety and higher BP risk [17, 18]. It is necessary to investigate the relationship between COVID-19-related anxiety and home BP changes in elderly patients with hypertension. Our previous study demonstrated that the fluctuation in BP in patients with hypertension is closely related to the severity of pandemic and emergency actions taken by the Chinese government [19]. The epidemic rapidly broke out and spread throughout China in January 2020, which greatly challenged the physical and mental health of the elderly. Recent studies have shown that psychological and behavioral responses to the pandemic were dramatic during the rising period of the COVID-19 outbreak, which led to a high prevalence of anxiety, especially in the Wuhan area [20]. Our data showed that during the COVID-19 outbreak, patients with anxiety had an increasing trend in average morning SBP compared with those who did not report anxiety, both in Wuhan and in other areas of China. Moreover, BP exhibited a seasonal variation in winter among patients without anxiety, but the seasonal BP variation among patients with anxiety was suppressed during the pandemic. A Japanese study also demonstrated that there was no significant seasonal BP variation in the first year after the disaster [21]. Consistently, a short-term pressor effect of the COVID-19 outbreak might overwhelm the seasonal BP variation. One potential explanation is that higher BP in anxious individuals reflects a chronic state of psychological arousal, which is typically accompanied by increased sympathetic nervous system activity and decreased parasympathetic activity. At the vascular level, increased norepinephrinergic activity may further increase peripheral resistance.

Clinical studies have shown that changes in SBP may be a prognostic surrogate marker for predicting cardiovascular outcomes [22, 23], and SBP control can greatly reduce the risk of cardiovascular events, including coronary heart disease, stroke, and heart failure [24]. Anxiety is considered an etiological factor in cardiovascular disease, although evidence on the association between anxiety and cardiovascular disease is contradictory. Recent evidence demonstrated that anxiety contributes to the development of coronary heart disease and cardiac mortality in a meta-analysis including 20 studies and 250 thousand persons with a mean follow-up period of 11.2 years [25], but several other studies found no association [26]. These differences could be partly explained by differences in study design and populations. In this study, we provided evidence that COVID-19-related anxiety was independently associated with poor prognosis of cardiovascular disease during the 1-year follow-up period in elderly patients with hypertension after adjustment for age, sex, BMI, education level, medication use, baseline SBP during the pre-epidemic period, and change in average morning SBP during the pandemic. Future research may focus on the mechanisms through which anxiety might affect cardiovascular disease.

One important strength of this study is that the smartphone-based app platform has been used to monitor home BP for patients. It can generate graphical representations of daily, weekly, and monthly home BP data during the follow-up period and facilitate more effective hypertension management. This study showed that patients paid more attention to self-monitoring of home BP during the specific health crisis, and the average number of BP checks by patients *via* the app showed an increasing trend compared with the pre-epidemic period. As expected, since doctors were engaged in combating the pandemic, they were less likely to monitor the app for BP control of patients, and the frequency of app visits per doctor fell substantially during the pandemic. In accordance, the rates of uncontrolled BP in patients with anxiety were higher than those in patients without anxiety. These data suggested that the app can be improved in the future by constructing a team-based care system that includes doctors and other practice providers (such as nurses, pharmacists, or physician assistants) to strengthen the efficiency of remote monitoring and BP management, particularly for elderly individuals who suffer from unstable BP or are at high risk.

Several major limitations in this study should be mentioned. First, the patient population was from a clinical trial cohort, and thus, the effects in other populations could be different, especially as these patients are under active treatment. Second, our available data permitted only a short-term assessment of cardiovascular events; further study is needed to assess the long-term effect of COVID-19-related anxiety on cardiovascular diseases in older patients with hypertension. Third, the sample of patients with anxiety in Wuhan (*n* = 13) used for subgroup analysis was small, leading to limited statistical power, and we also lacked home BP data from clinic centers in Hubei Province except Wuhan City (the pandemic center). Thus, our study may not be representative of anxiety status in areas under severe situations, such as traffic and behavioral restrictions during the pandemic. Finally, we surveyed the health status of patients at only one time point during the outbreak. Anxiety fluctuates over time, so attempts to categorize individuals as anxious or not on the basis of a single assessment may be unreliable [27]. In addition, patients’ anxiety symptoms were assessed using sum scores of the validated GAD-7 questionnaire; such scores are not comparable with a diagnosis of general anxiety based on a clinical interview. Repeated measurements of anxiety status would be helpful to determine how anxiety has affected BP variation and cardiovascular risk during the pandemic.

## Conclusions

In summary, our data provide evidence that COVID-19-related anxiety is associated with a short-term increase in home morning SBP among older patients and leads to a greater risk of cardiovascular events during the 1-year follow-up period. Epidemic prevention and control measures should focus more on mental health care for older patients, and future studies are needed to assess the long-term effect of anxiety on cardiovascular risk in other populations in the setting of a pandemic crisis.

## Supplementary information

Online Materials
