# Supplementary material for: Anxiety, home blood pressure monitoring, and cardiovascular events among older hypertension patients during the COVID-19 pandemic
Source: Hypertens Res. 2022 Jan 21;45(5):856–65. doi: 10.1038/s41440-022-00852-0 (PMC8778505; doi:10.1038/s41440-022-00852-0)
Supplement: Supplementary file 1 — Online Materials [file 41440_2022_852_MOESM1_ESM.docx]

**Online Materials**

**Full title:** Anxiety, home blood pressure monitoring, and cardiovascular events among older hypertension patients during COVID-19 pandemic

**Online Methods**

**Online Tables**

Supplementary Table 1. Comparison of characteristics at the pre-epidemic period between the included and excluded patients in this study

Supplementary Table 2. Clinical characteristics at the pre-epidemic period in patients with anxiety or without anxiety in Wuhan and non-Wuhan areas of China

Supplementary Table 3. Change of average morning SBP in patients with anxiety or without anxiety stratified by Wuhan and non-Wuhan areas during the COVID-19 period

Supplementary Table 4. Rate of uncontrolled BP in patients with anxiety or without anxiety during the COVID-19 period

Supplementary Table 5. The morning SBP variability in patients with anxiety or without anxiety during the COVID-19 period

Supplementary Table 6. Change in average morning DBP in patients with anxiety or without anxiety during the COVID-19 period

Supplementary Table 7. Use of antihypertensive medication in patients with anxiety or without anxiety during the COVID-19 period

Supplementary Table 8. Patients attending online lectures in relation to average morning SBP during the COVID-19 period

Supplementary Table 9. Change in physical activities in relation to average morning SBP during the COVID-19 period

**Online Figures**

Supplementary Figure 1. Timeline of the COVID-19 outbreak in Wuhan and other provinces of China

Supplementary Figure 2. Geographical distribution of 42 participating hospitals in this study

Supplementary Figure 3. Average morning SBP and numbers of BP measurement per week in patients with anxiety or without anxiety during the COVID-19 period

Supplementary Figure 4. Trajectory pattern of average morning SBP in patients with anxiety or without anxiety stratified by Wuhan and non-Wuhan areas during the COVID-19 period

Supplementary Figure 5. Seasonal variation in winter of average morning SBP in older patients in the previous year (November 20, 2018 to March 21, 2019)

Supplementary Figure 6. Fluctuations of average morning SBP in relation to the frequency of app visits by doctors during the COVID-19 period

Supplementary Figure 7. Fluctuations of average morning SBP in relation to the frequency of app visits by patients during the COVID -19 period

**Appendix 1.** Questionnaire for health status of elderly patients with hypertension during the COVID-19 pandemic

**Online methods**

**Full list of participating hospitals and principal investigators in the STEP study**

FuWai Hospital, Beijing, Jun Cai, Weili Zhang

Bei Jing Hospital, Beijing, Wei Liu

Peking Union Medical College Hospital, Beijing, Anli Tong

Beijing Chaoyang Hospital affiliated to Capital Medical University, Beijing,

Xinchun Yang, Qianmei Sun

The Hospital of Shunyi District, Beijing, Hongwei Zhang

Beijing Pinggu Hospital, Beijing, Yufeng Li

Pingjin Hospital, Logistics University of PAPF, Tianjin, Yuming Li

The People's Hospital of Ji Xian District, Tianjin, Jinfeng Yang

Kailuan General Hospital, Tangshan, Hebei, Shouling Wu

The First Affiliated Hospital of Hebei North University, Zhangjiakou, Hebei, Lixin Wang

Shanxi Bethune Hospi tal, Shanxi Academy of Medical Sciences, Taiyuan, Shanxi, Jie Ren

First Hospital of Shanxi Medical University, Taiyuan, Shanxi, Xiaomei Shen

Shanxi Cardiovascular Hospital, Taiyuan, Shanxi, Liangqing Zhang

The Second Affiliated Hospital of Baotou Medical College, Baotou, Inner Mongolia,

Gang Sun

The First People's Hospital of Yinchuan, Yinchuan, Ningxia, Fang Chen

Benxi Railway Hospital, Beixi, Liaoning, Lianchen Yao

First Affiliated Hospital of Harbin Medical University, Harbin, Heilongjiang, Xinhua Yin

Hongxinglong Center Hospital, Shuangyashan, Heilongjiang, Dianfang Li

The 1st Affiliated Hospital of Dalian Medical University, Dalian, Liaoning, Yinong Jiang

Lanzhou University Second Hospital, Lanzhou, Gansu, Jing Yu

First Affiliated Hospital of Xinjiang Medical University, Urumqi, Xinjiang, Xinjuan Xu

First Affiliated Hospital, Xian Jiaotong University, Xi'an, Shanxi, Gang Tian

Qilu Hospital of Shandong University, Jinan, Shandong, Peili Pu, Changjie Ren

Shanghai General Hospital, Shanghai Jiaotong University, Shanghai, Qiuyan Dai

Zhenjiang First People's Hospital, Zhenjiang, Jiangsu, Guohui Zhang

Wuhan Renmin Hospital of Wuhan University, Wuhan, Hubei, Xiaoyang Zhou

Zhoukou City Central Hospital, Zhoukou, Henan, Hualing Liu

First Affiliated Hospital of Zhengzhou University, Zhengzhou, Henan, Luosha Zhao

Kang Ya Hospital, Yiyang, Hunan, Bingpo Zhu

The Second Affiliated Hospital of NanChang University, Nanchang, Jiangxi, Ping Li, Yanhua Tao

Guangdong Cardiovascular Institute, Guangzhou, Guangdong, Yingqing Feng

Huizhou Municipal Central Hospital, Huizhou, Guangdong, Jinguang Liu

The Second Affiliated Hospital to Medical College Shantou University, Shantou, Guangdong, Youren Chen

Shenzhen Sun Yat-sen Cardiovascular Hospital, Shenzhen, Guangdong, Xiaoyuan Tian

The First Affiliated Hospital of Guangxi University of Chinese Medicine, Nanning, Guangxi, Chaoxin Pan

The First Affiliated Hospital of Guangxi Medical University, Nanning, Guangxi, Rongjie Huang

West China Hospital, Sichuan University, Chengdu, Sichuan, Xiaoping Chen, Xinjun Zhang

Yunnan Cardiovascular Hospital, Kunming, Yunnan, Li Yang

The Second Affiliated Hospital of Kunming Medical University, Kunming, Yunnan, Zihong Guo

The First Hospital of Kunming, Kunming, Yunnan, Yunlan Liu

College of Medicine, National Taiwan University, Taipei, Taiwan, Tzung-Dau Wang

**Inclusion and exclusion criteria**

**Inclusion criteria:**

Subjects were eligible to be included in the trial only if all of the following criteria apply:

1. Systolic blood pressure (SBP) between 140−190 mm Hg in the three screening visits or currently under anti-hypertension treatment.

2. An age of 60−80 years, Han ethnicity.

3. Signed the written informed consent.

**Exclusion criteria:**

Subjects were excluded from the trial if any of the following criteria apply:

1. SBP ≥ 190 mm Hg, or diastolic blood pressure (DBP) < 60 mm Hg.

2. Diagnosed secondary hypertension.

3. History of large atherosclerotic cerebral infarction or hemorrhagic stroke (not including lacunar infarction and transient ischemic attack [TIA]).

4. Hospitalization for myocardial infarction (MI) within the last 6 months, but remote MI events were included.

5. Coronary revascularization (percutaneous coronary intervention [PCI] or coronary artery bypass grafting [CABG]) within the last 12 months.

6. Planned to perform PCI or CABG in the next 12 months.

7. History of sustained atrial fibrillation or ventricular arrhythmias at entry influencing the measurement of electronic blood pressure.

8. New York Heart Association (NYHA) class III-IV heart failure or hospitalization for exacerbation of chronic heart failure at entry.

9. Severe valvular disease or valvular disease likely to require surgery or percutaneous valve replacement during the trial.

10. Hypertrophic cardiomyopathy (HCM), a disease characterized by unexplained left ventricular (LV) hypertrophy associated with non-dilated ventricular chambers in the absence of another cardiac or systemic disease. HCM is usually recognized by maximal LV wall thickness ≥ 15 mm unexplained by abnormal loading conditions, or with wall thickness of 13 to 14 mm considered borderline, particularly in the presence of other compelling information, e.g. family history of HCM, based on echocardiography. In older patients with LV hypertrophy and a history of long-standing systemic hypertension, the HCM can be determined by identification of a diagnostic sarcomere mutation or inferred by marked LV thickness > 25 mm and/or left ventricular outflow tract obstruction with systolic anterior motion and mitral-septal contact ^[1]^.

11. Dilated cardiomyopathy, rheumatic heart disease, or congenital heart disease ^[2]^.

12. Uncontrolled diabetes mellitus (serum fasting glucose ≥ 200 mg/dl [11.1 mmol/L], glycated hemoglobin [HbA1] > 8%).

13. Severe liver or kidney dysfunction (Alanine aminotransferase [ALT] ≥ 3 times the upper limit of normal value, or end stage renal disease on dialysis or estimated glomerular filtration rate [eGFR] < 30 mL/min/1.73 m^2^, or serum creatinine > 2.5 mg/dL [> 221 μmol/L]).

14. Severe somatic disease such as cancer.

15. Severe cognitive impairment or mental disorders.

16. Participating in other clinical trials.

**Definition and ascertainment of outcomes**

The primary cardiovascular outcome of the STEP trial was a composite of the first occurrence of acute coronary syndrome (myocardial infarction and hospitalization for unstable angina), stroke (ischemic or hemorrhagic, fatal or nonfatal), acute decompensated heart failure, coronary revascularization (PCI or CABG), atrial fibrillation, or death from cardiovascular causes. The definition and ascertainment of outcomes were as the following:

1. **Myocardial infarction.** The diagnosis of myocardial infarction is based on the following criteria ^[3]^: (1) Patient has cardiac signs and symptoms, such as retrosternal pain last for at least 30 minutes, and not relieve to nitroglycerine during the attack; (2) Electrocardiographic abnormal findings of MI are observed; (3) Biochemical markers of cardiac damage are present.

2. **Hospitalization for unstable angina.** The diagnosis of unstable angina requires hospitalization for evaluation. The clinical presentation of unstable angina includes: (1) prolonged (> 20 min) angina pain at rest; (2) new onset angina; (3) post-MI angina; (4) recent destabilization of previously stable angina with at least Canadian Cardiovascular Society Class III angina characteristics ^[4]^.

3. **Hospitalization for acute decompensated heart failure.** Diagnosis of acute decompensated heart failure requires a hospitalization or emergency department visit which provides an infusion therapy for clinical signs and symptoms consistent with cardiac decompensation or inadequate cardiac pump function, such as increasing or new onset shortness of breath, peripheral edema, paroxysmal dyspnea, orthopnea, or hypoxia ^[5,6]^.

4. **Cardiovascular death.** Cardiovascular death includes fatal coronary heart disease, fatal stroke, death from heart failure, and sudden cardiac death.

5. **Atrial fibrillation.** The diagnosis requires rhythm evidence using an electrocardiogram (ECG) showing the typical pattern of atrial fibrillation: absolutely irregular RR intervals and no discernible, distinct P waves ^[7]^.

6. **First occurrence of symptomatic stroke (ischemic or hemorrhagic stroke).** Stroke is defined as a rapid onset of focal (or global) disturbance of cerebral function lasting >24 hours (except interrupted by surgery or death) without resolution of symptoms according to the World Health Organization ^[8]^. The diagnosis of stroke is confirmed by strict neurological examination, computed tomography (CT), or magnetic resonance imaging (MRI), and stroke subtypes are classified including ischemic or hemorrhagic ^[9,10]^.

Atherothrombotic stroke was defined as an occlusion or > 50% stenosis of a major brain artery or branch cortical artery on duplex sonography or angiography and corresponding to the clinical features of cerebral cortical, brain stem or cerebellar dysfunction; no definite cardiac source of embolism; cortical, cerebellar, brain stem or subcortical infarct was ≥ 15 mm in diameter on CT or MRI.

Hemorrhagic stroke is diagnosed if a CT scan demonstrated an area of hyperdensity within the brain parenchyma with or without extension into the ventricles or subarachnoid space or, for scans performed beyond 1 week, an area of attenuation with ring enhancement after injection of contrast.

The definition of a stroke does not include transient ischemic attack (TIA) and lacunar infarction, but they are recorded during the course of the trial.

**Antihypertensive drugs treatment**

In this study, represented antihypertensive drugs were provided to the patients at no cost, including angiotensin receptor blockers (ARBs), calcium channel blockers (CCBs) and thiazide-type diuretics, all of which have robust evidence to reduce BP and prevent cardiovascular events. For all participants, olmesartan medoxomil tablets (Nanjing Chia Tai Tianqing Pharmaceutical Co., Ltd, Nanjing, China) was the preferred ARBs at a daily dose of 20 mg (once daily), and amlodipine besylate tablet (China Resources Saike Pharmaceutical Co., Ltd, Beijing, China) as the preferred CCBs at a daily dose of 5–10 mg (once daily). Hydrochlorothiazide was not designed as the initial therapy. Other antihypertensive drugs such as beta-adrenergic blockers (β-blockers) may also be used when an investigator deems necessary and appropriate. The investigators are allowed to prescribe an individualized antihypertensive regimen regarding which drugs to use at initiation and during the intensive treatment in order to achieve and maintain SBP targets.

**References:**

1. Gersh BJ, Maron BJ, Bonow RO, et al. 2011 ACCF/AHA guideline for the diagnosis and treatment of hypertrophic cardiomyopathy: a report of the American College of Cardiology Foundation/American Heart Association Task Force on Practice Guidelines. *Circulation* 2011; 124: e783–e831.
2. Elliott P, Andersson B, Arbustini E, et al. Classification of the cardiomyopathies: a position statement from the European Society of Cardiology Working Group on Myocardial and Pericardial Diseases. *Eur Heart J* 2008; 29: 270–276.
3. Luepker RV, Apple FS, Christenson RH, et al. Case definitions for acute coronary heart disease in epidemiology and clinical research studies: a statement from the AHA Council on Epidemiology and Prevention; AHA Statistics Committee; World Heart Federation Council on Epidemiology and Prevention; the European Society of Cardiology Working Group on Epidemiology and Prevention; Centers for Disease Control and Prevention; and the National Heart, Lung, and Blood Institute. *Circulation* 2003; 108: 2543–2549.
4. Hamm CW, Bassand JP, Agewall S, et al. ESC Guidelines for the management of acute coronary syndromes in patients presenting without persistent ST-segment elevation: The Task Force for the management of acute coronary syndromes (ACS) in patients presenting without persistent ST-segment elevation of the European Society of Cardiology (ESC). *Eur Heart J* 2011; 32: 2999−3054.
5. Rosamond WD, Chang PP, Baggett C, et al. Classification of heart failure in the atherosclerosis risk in communities (ARIC) study: a comparison of diagnostic criteria. *Circ Heart Fail* 2012; 5: 152−159.
6. Loehr LR, Agarwal SK, Baggett C, et al. Classification of acute decompensated heart failure: an automated algorithm compared with a physician reviewer panel: the Atherosclerosis Risk in Communities study. *Circ Heart Fail* 2013; 6: 719−726.
7. Kirchhof P, Benussi S, Kotecha D, *et al*. 2016 ESC Guidelines for the management of atrial fibrillation developed in collaboration with EACTS. *Europace* 2016; 18: 1609−1678.
8. World Health Organization. International classification of diseases. 10th revision. Geneva: World Health Organization, 1993.
9. Adams HJ, Bendixen BH, Kappelle LJ, et al. Classification of subtype of acute ischemic stroke. Definitions for use in a multicenter clinical trial. TOAST. Trial of Org 10172 in Acute Stroke Treatment. *Stroke* 1993; 24: 35−41.
10. Easton JD, Saver JL, Albers GW, et al. Definition and evaluation of transient ischemic attack: a scientific statement for healthcare professionals from the American Heart Association/American Stroke Association Stroke Council; Council on Cardiovascular Surgery and Anesthesia; Council on Cardiovascular Radiology and Intervention; Council on Cardiovascular Nursing; and the Interdisciplinary Council on Peripheral Vascular Disease. The American Academy of Neurology affirms the value of this statement as an educational tool for neurologists. *Stroke* 2009; 40: 2276−2293.

**Supplementary Table 1. Comparison of characteristics at the pre-epidemic period between the included and excluded patients in this study**

| **Characteristics** | **Included (n=3724)** | **Excluded (n=4787)** | ***P* value*** |
| --- | --- | --- | --- |
| Age, years | 68.3 ± 4.7 | 69.0 ± 5.0 | <0.001 |
| Men, No. (%) | 1731 (46.5) | 2228 (46.6) | 0.96 |
| Body mass index, kg/m^2^ | 25.7 ± 3.2 | 25.5 ± 3.1 | 0.03 |
| Morning SBP, mm Hg | 132 ± 10 | 133 ± 10 | 0.18 |
| Morning DBP, mm Hg | 80 ± 8 | 81 ± 7 | 0.17 |
| Fasting glucose, mmol/L | 6.1 ± 1.6 | 6.2 ± 1.8 | 0.10 |
| Lipids profile, mmol/L |  |  |  |
| Total cholesterol | 4.9 ± 1.1 | 4.9 ± 1.2 | 0.36 |
| Triglycerides | 1.3 (1.0–1.9) | 1.4 (1.0–2.0) | 0.11 |
| HDL-C | 1.3 ± 0.3 | 1.3 ± 0.3 | 0.91 |
| LDL-C | 2.7 ± 0.9 | 2.7 ± 0.9 | 0.59 |
| Educational level, No. (%) |  |  |  |
| Middle school or below | 1970 (52.9) | 2864 (59.9) | <0.001 |
| High school or above | 1754 (47.1) | 1919 (40.1) |  |
| Smoking status, No. (%) |  |  |  |
| Never | 2664 (71.7) | 3435 (72.0) | 0.66 |
| Former | 458 (12.3) | 558 (11.7) |  |
| Current | 594 (16.0) | 777 (16.3) |  |
| Alcohol intake, No. (%) |  |  |  |
| Never | 2519 (67.8) | 3277 (68.7) | 0.21 |
| Former | 185 (5.0) | 263 (5.5) |  |
| Current | 1012 (27.2) | 1228 (25.8) |  |
| Medical history, No. (%) |  |  |  |
| Diabetic mellitus | 728 (19.5) | 898 (18.8) | 0.37 |
| Coronary heart disease | 198 (5.3) | 342 (7.1) | 0.001 |
| The 10-year risk of cardiovascular disease ≥15%, No. (%)^†^ | 2368 (63.9) | 3032 (65.1) | 0.25 |

Abbreviations: SBP, systolic blood pressure; DBP, diastolic blood pressure; HDL-C, high-density lipoprotein cholesterol; LDL-C, low-density lipoprotein cholesterol.

Values were given as mean ± SD, number (%), or median (interquartile range).

^*^*P* values were calculated by Student *t* test or Mann-Whitney nonparametric test for quantitative variables, or by Chi-square test for qualitative variables, when appropriate.

^†^ The 10-year CVD risk was estimated by Framingham risk score, and patients with a ≥15% risk score were considered at high-risk.

**Supplementary Table 2. Clinical characteristics at the pre-epidemic period in patients with anxiety or without anxiety in Wuhan and non-Wuhan areas of China**

|  | **In Wuhan (n=240)** | | | **In non-Wuhan areas (n=3484)** | | |
| --- | --- | --- | --- | --- | --- | --- |
| **Characteristics** | **Without anxiety**  **(n=227)** | **With anxiety (n=13)** | ***P* ^*^** | **Without anxiety**  **(n=3235)** | **With anxiety (n=249)** | ***P*** ^*^ |
| Age, years | 68.3 ± 4.7 | 69.8 ± 6.9 | 0.25 | 68.3 ± 4.7 | 68.6 ± 4.6 | 0.24 |
| Men, No. (%) | 117 (51.5) | 6 (46.2) | 0.53 | 1494 (46.2) | 114 (45.8) | 0.90 |
| Body mass index, kg/m^2^ | 25.5 ± 3.5 | 24.9 ± 3.6 | 0.53 | 25.7 ± 3.2 | 25.7 ± 3.2 | 0.71 |
| Morning SBP, mm Hg | 131.7 **±** 9.4 | 134.8 ± 11.6 | 0.25 | 131.9 **±** 9.5 | 130.9 ± 9.2 | 0.11 |
| Fasting glucose, mmol/L | 6.0 ± 1.5 | 5.5 ± 1.2 | 0.31 | 6.2 ± 1.6 | 6.3 ± 2.0 | 0.12 |
| Lipid profile, mmol/L |  |  |  |  |  |  |
| Total cholesterol | 4.8 ± 1.0 | 5.1 ± 1.8 | 0.33 | 4.9 ± 1.1 | 5.0 ± 1.1 | 0.51 |
| Triglycerides | 1.2 (0.8–1.8) | 1.6 (0.8–1.8) | 0.51 | 1.3 (1.0–1.9) | 1.4 (1.0–2.0) | 0.23 |
| HDL-C | 1.3 ± 0.3 | 1.3 ± 0.3 | 0.73 | 1.3 ± 0.3 | 1.3 ± 0.3 | 0.82 |
| LDL-C | 2.7 ± 0.8 | 2.6 ± 1.7 | 0.94 | 2.7 ± 0.9 | 2.7 ± 1.0 | 0.85 |
| Educational level, No. (%) |  |  |  |  |  |  |
| Middle school or below | 147 (64.8) | 5 (38.4) | 0.03 | 1687 (52.1) | 132 (53.0) | 0.79 |
| High school or above | 80 (35.2) | 8 (61.5) |  | 1548 (47.9) | 117 (47.0) |  |
| Smoking status, No. (%) |  |  |  |  |  |  |
| Never | 150 (66.7) | 10 (76.9) | 0.54 | 2316 (71.7) | 188 (75.5) | 0.12 |
| Former | 33 (14.7) | 2 (14.3) |  | 390 (12.1) | 33 (13.3) |  |
| Current | 42 (18.7) | 1 (7.7) |  | 523 (16.2) | 28 (11.2) |  |
| Alcohol intake, No. (%) |  |  |  |  |  |  |
| Never | 142 (63.1) | 10 (76.9) | 0.44 | 2195 (68.0) | 172 (69.1) | 0.89 |
| Former | 11 (4.9) | 0 (0) |  | 163 (5.0) | 11 (4.4) |  |
| Current | 72 (32.0) | 3 (23.1) |  | 871 (27.0) | 66 (26.5) |  |
| Medical history, No. (%) |  |  |  |  |  |  |
| Diabetic mellitus | 50 (22.0) | 5 (38.4) | 0.24 | 624 (19.3) | 50 (20.1) | 0.76 |
| Coronary heart disease | 6 (2.6) | 1 (7.7) | 0.33 | 178 (5.5) | 13 (5.2) | 0.85 |
| The 10-year risk of CVD, %^†^ | 19.5 ± 8.6 | 19.4 ± 7.8 | 0.96 | 19.2 ± 8.4 | 19.4 ± 8.8 | 0.63 |
| The 10-year risk of CVD ≥15%, No. (%)^†^ | 149 (66.2) | 10 (76.9) | 0.69 | 2050 (63.7) | 160 (64.5) | 0.80 |
| Antihypertensive medications, No. (%) |  |  |  |  |  |  |
| Calcium channel blocker | 186 (83.4) | 11 (84.5) | 0.40 | 2474 (83.2) | 196 (85.6) | 0.35 |
| Angiotensin receptor blocker | 170 (76.2) | 11 (84.5) | 0.18 | 2338 (78.6) | 189 (82.5) | 0.16 |
| Beta-blocker | 8 (3.6) | 1 (7.7) | 0.45 | 164 (5.5) | 10 (4.4) | 0.46 |
| Hydrochlorothiazide | 43 (19.3) | 3 (23.1) | 0.31 | 360 (12.1) | 34 (14.8) | 0.22 |

Abbreviations: SBP, systolic blood pressure; HDL-C, high-density lipoprotein cholesterol; LDL-C, low-density lipoprotein cholesterol; CVD, cardiovascular diseases.

Values were given as mean ± SD, number (%), or median (interquartile range).

^*^ *P* values were calculated for comparison between patents with and without anxiety.

^†^ The 10-year CVD risk was estimated by Framingham risk score.

**Supplementary Table 3. Change of average morning SBP in patients with anxiety or without anxiety stratified by Wuhan and non-Wuhan areas during the COVID-19 period**

| **The COVID-19 period** | **Adjusted mean (95%CI) of SBP, mm Hg*** | |  |  | **Adjusted mean difference (95%CI)**  **of SBP (∆SBP), mm Hg**^‡^ | | **The between-group difference in ∆SBP** | ***P***^§^ |
| --- | --- | --- | --- | --- | --- | --- | --- | --- |
|  | **Without anxiety**  **(GAD-7≤4)** | **With anxiety**  **(GAD-7≥5)** | ***P* *** |  | **Without anxiety**  **(GAD-7≤4)** | **With anxiety**  **(GAD-7≥5)** |  |  |
| **In Wuhan (n=240)** | 227 | 13 |  |  |  |  |  |  |
| Pre-epidemic period | 131.8 (130.3 to 133.2) | 134.8 (129.1 to 140.5) | 0.31 |  | **-** | - | - |  |
| Incubation period | 134.1 (132.6 to 135.6) ^†^ | 137.2 (131.0 to 143.3) | 0.34 |  | 2.7 (1.7 to 3.8) | 1.5 (-3.6 to 5.8) | -1.6 (-6.4 to 3.2) | 0.51 |
| Developing period | 135.2 (133.7 to 135.2) ^†^ | 136.3 (129.9 to 142.6) | 0.74 |  | 3.8 (2.4 to 5.1) | 0.7 (-5.4 to 6.8) | -3.0 (-9.3 to 3.2) | 0.34 |
| Outbreak period | 133.9 (132.3 to 135.5) ^†^ | 136.0 (129.2 to 142.8) | 0.56 |  | 2.1 (0.7 to 3.5) | 1.0 (-5.9 to 7.9) | -1.1 (-8.1 to 6.0) | 0.76 |
| Plateau period | 131.1 (129.6 to 132.6) | 134.7 (128.7 to 147.0) | 0.25 |  | -0.7 (-2.1 to 0.7) | -0.9 (-7.1 to 5.3) | -0.1 (-6.5 to 6.2) | 0.96 |
| **In non-Wuhan areas (n=3484)** | 3235 | 249 |  |  |  |  |  |  |
| Pre-epidemic period | 130.9 (130.6 to 131.2) | 131.9 (130.7 to 133.0) | 0.12 |  | - | - | - |  |
| Incubation period | 131.1 (130.8 to 131.5) ^†^ | 132.5 (131.3 to 133.7) | 0.03 |  | 0.2 (0.03 to 0.4) | 0.5 (-0.2 to 1.3) | 0.3 (-0.4 to 1.1) | 0.41 |
| Developing period | 131.3 (131.0 to 131.6) ^†^ | 132.7 (131.5 to 133.9) ^†^ | 0.02 |  | 0.4 (0.1 to 0.6) | 0.8 (-0.1 to 1.6) | 0.4 (-0.5 to 1.3) | 0.37 |
| Outbreak period | 130.5 (130.2 to 130.9) ^†^ | 132.8 (131.6 to 134.0) ^†^ | <0.001 |  | -0.4 (-0.6 to -0.1) | 0.9 (0.02 to 1.9) | 1.3 (0.4 to 2.3) | 0.007 |
| Plateau period | 129.9 (129.6 to 130.3) ^†^ | 132.7 (131.5 to 133.8) ^†^ | <0.001 |  | -1.0 (-1.2 to -0.7) | 0.8 (-0.1 to 1.7) | 1.8 (0.8 to 2.7) | <0.001 |

Abbreviations: SBP, systolic blood pressure; COVID-19, coronavirus disease 2019; CI, confidence interval; GAD-7, generalized anxiety disorder scale-7.

The pandemic timeline of the COVID-19 in China was classified as the pre-epidemic period as the reference (October 21 to November 20, 2019), incubation period (November 21 to December 20, 2019), developing period (December 21, 2019 to January 20, 2020), outbreak period (January 21 to February 20, 2020), and plateau period (February 21 to March 21, 2020). **^*^**Adjusted mean (95%CI) of SBP was calculated by linear mixed model after adjustment for age, sex, and BMI, and *P* value was compared between patients with anxiety (GAD-7 ≤4) and without anxiety (GAD-7 ≥5).

^†^*P*<0.05, each period of epidemic *versus* the pre-epidemic period (as the reference group), calculated by linear mixed model adjusting for age, sex, and BMI.

^‡^Adjusted mean difference (95%CI) of SBP (∆SBP) was calculated as the change of average morning SBP from pre-epidemic to each time period of COVID-19.

^§^*P* value was compared between patients with anxiety and without anxiety by linear regression model after adjustment for age, sex, and BMI.

**Supplementary Table 4. Rate of uncontrolled BP in patients with anxiety or without anxiety during the COVID-19 period**

|  | **Rate of uncontrolled BP (≥140/90 mm Hg), no (%)** | | ***P*** |
| --- | --- | --- | --- |
| **The COVID-19 period** | **In patients without anxiety**  **(GAD-7≤4)** | **In patients with anxiety**  **(GAD-7≥5)** | **value**^*^ |
| No. of patients | 3462 | 262 |  |
| Pre-epidemic period  (Oct 21^st^ – Nov 20^th^, 2019) | 740 (21.4%) | 71 (27.1%) | 0.03 |
| Incubation period  (Nov 21^st^ – Dec 20^th^, 2019) | 707 (20.4%) ^†^ | 61 (23.3%) ^†^ | 0.02 |
| Developing period  (Dec 21^st^, 2019 – Jan 20^th^, 2020) | 721 (20.8%) ^†^ | 57 (21.8%) ^†^ | 0.02 |
| Outbreak period  (Jan 21^st^ – Feb 20^th^, 2020) | 639 (18.5%) ^†^ | 57 (21.8%) ^†^ | 0.01 |
| Plateau period  (Feb 21^st^ – Mar 21^st^, 2020) | 640 (18.5%) ^†^ | 78 (29.8%) | <0.001 |

Abbreviations: BP, blood pressure; COVID-19, coronavirus disease 2019; GAD-7, generalized anxiety disorder scale-7.

^*^*P* value was calculated by Chi-square test.

^†^*P* <0.001, the pre-epidemic period considered as the reference group, calculated by Cochran-Armitage trend test.

**Supplementary Table 5. The morning SBP variability in patients with anxiety or without anxiety during the COVID-19 period**

|  | **Coefficient of variation of morning SBP^*^** | | |  | **Difference in morning SBP^*^** | | |
| --- | --- | --- | --- | --- | --- | --- | --- |
| **The COVID-19 period** | **Without anxiety**  **(GAD-7≤4)** | **With anxiety**  **(GAD-7 ≥5)** | ***P* value**^†^ |  | **Without anxiety**  **(GAD-7≤4)** | **With anxiety**  **(GAD-7 ≥5)** | ***P* value**^†^ |
| No. of patients | 3462 | 262 |  |  | 3462 | 262 |  |
| Pre-epidemic period | 5.1% (0.02) | 5.1% (0.02) | 0.94 |  | 23.6 (12.5) | 23.3 (13.5) | 0.74 |
| Incubation period | 5.0% (0.02) | 5.0% (0.02) | 0.64 |  | 23.4 (12.3) | 22.6 (13.0) | 0.32 |
| Developing period | 5.0% (0.02) | 4.9% (0.02) | 0.78 |  | 23.2 (12.5) | 23.4 (13.8) | 0.90 |
| Outbreak period | 4.9% (0.02) | 4.7% (0.03) | 0.21 |  | 22.6 (12.7) | 22.6 (14.0) | 0.99 |
| Plateau period | 4.8% (0.02) | 4.9% (0.03) | 0.80 |  | 22.5 (12.2) | 22.3 (12.5) | 0.79 |

Abbreviations: COVID-19, coronavirus disease 2019; SBP, systolic blood pressure; GAD-7, generalized anxiety disorder scale-7.

Data was expressed as mean (SD).

^*^ SBP variability was evaluated by the coefficient of variation of morning SBP (expressed as standard deviation /mean×100%) and the difference in morning SBP (maximum - the minimum in morning SBP) at each period of the pandemic.

^†^*P* value was calculated by Student-*t* test.

**Supplementary Table 6. Change in average morning DBP in patients with anxiety or without anxiety during the COVID-19 period**

| **The COVID-19 period** | **Adjusted mean (95%CI) of DBP, mm Hg*** | |  |  | **Adjusted mean difference (95%CI) of DBP (∆DBP), mm Hg**^‡^ | | **The between-group difference in ∆DBP** | ***P***^§^ |
| --- | --- | --- | --- | --- | --- | --- | --- | --- |
|  | **Without anxiety (GAD-7≤4)**  **(n=3462)** | **With anxiety**  **(GAD-7≥5)**  **(n=262)** | ***P* *** |  | **Without anxiety**  **(GAD-7≤4)**  **(n=3462)** | **With anxiety**  **(GAD-7≥5)**  **(n=262)** |  |  |
| Pre-epidemic period | 79.8 (79.5, 80.0) | 80.0 (79.1, 80.9) | 0.26 |  |  |  |  |  |
| Incubation period | 79.7 (79.5, 80.0) | 80.3 (79.4, 81.2) | 0.22 |  | 0.01 (-0.1, 0.1) | 0.3 (-0.1, 0.7) | 0.3 (-0.2, 0.7) | 0.21 |
| Developing period | 79.8 (79.5, 80.0) | 80.3 (79.3, 81.2) | 0.32 |  | 0.04 (-0.1, 0.2) | 0.2 (-0.3, 0.6) | 0.1 (-0.4, 0.6) | 0.70 |
| Outbreak period | 79.5 (79.2, 79.7) ^†^ | 80.3 (79.3, 81.3) | 0.13 |  | -0.3 (-0.4, -0.1) | 0.3 (-0.3, 0.8) | 0.5 (-0.02, 1.1) | 0.06 |
| Plateau period | 79.4 (79.1, 79.6) ^†^ | 80.6 (79.7, 81.6) ^†^ | 0.008 |  | -0.4 (-0.6, -0.3) | 0.5 (-0.01, 1.1) | 1.0 (0.4, 1.5) | 0.001 |

Abbreviations: DBP, diastolic blood pressure; COVID-19, coronavirus disease 2019; CI, confidence interval; GAD-7, generalized anxiety disorder scale-7.

The pandemic timeline of the COVID-19 in China was classified as mentioned above.

**^*^**Adjusted mean (95%CI) of DBP was calculated by linear mixed model after adjustment for age, sex, and BMI, and *P* value was compared between patients without anxiety and patients with anxiety.

^†^*P*<0.05, each period of epidemic *versus* the pre-epidemic period (as the reference group), calculated by linear mixed model adjusting for age, sex, and BMI.

^‡^Adjusted mean difference (95%CI) of DBP (∆DBP) was calculated as the change of average morning DBP from pre-epidemic period to each time period of COVID-19.

^§^*P* value was compared between patients with anxiety and without anxiety by linear regression model after adjustment for age, sex, and BMI.

**Supplementary Table 7.** **Use of antihypertensive medication in** **patients with anxiety or without anxiety during the COVID-19 period**

|  | **the COVID-19 period*** | | | | |
| --- | --- | --- | --- | --- | --- |
| **Variables** | **Pre-epidemic period** | **Incubation**  **period** | **Developing**  **period** | **Outbreak**  **period** | **Plateau**  **period** |
| **Patients without anxiety (n=3462)** | |  |  |  |  |
| Patients grouped by kinds of antihypertensive drugs, no. (%) |  |  |  |  |  |
| 1 | 1107 (35%) | 1097 (34%) | 1087 (34%) | 1064 (33%) | 1051 (33%) |
| 2 | 1650 (52%) | 1663 (52%) | 1671 (52%) | 1682 (53%) | 1694 (53%) |
| ≥3 | 439 (14%) | 436 (14%) | 438 (14%) | 450 (14%) | 451 (14%) |
| Patients grouped by class of antihypertensive drugs, no. (%) |  |  |  |  |  |
| Calcium channel blocker | 2673 (84%) | 2679 (84%) | 2676 (84%) | 2684 (84%) | 2690 (84%) |
| Angiotensin receptor blocker | 2512 (79%) | 2511 (79%) | 2521 (79%) | 2541 (80%) | 2546 (80%) |
| Beta-blocker | 172 (5%) | 167 (5%) | 174 (5%) | 181 (6%) | 182 (6%) |
| Hydrochlorothiazide | 423 (13%) | 422 (13%) | 422 (13%) | 428 (13%) | 433 (14%) |
| Patients grouped by medication changes, no. (%) |  | 123 (4%) | 406 (13%) | 616 (20%) | 645 (20%) |
| Increase of dosage or agent | – | 82 (3%) | 244 (8%) | 369 (12%) | 393 (12%) |
| Decrease of dosage or agent | – | 41 (1%) | 162 (5%) | 247 (8%) | 252 (8%) |
| **Patients with anxiety (n=262)** |  |  |  |  |  |
| Patients grouped by kinds of antihypertensive drugs, no. (%) |  |  |  |  |  |
| 1 | 69 (29%) | 69 (29%) | 75 (31%) | 74 (31%) | 75 (31%) |
| 2 | 132 (55%) | 133 (55%) | 129 (53%) | 132 (55%) | 130 (54%) |
| ≥3 | 41 (17%) | 40 (17%) | 38 (16%) | 36 (15%) | 36 (15%) |
| Patients grouped by class of antihypertensive drugs, no. (%) |  |  |  |  |  |
| Calcium channel blocker | 210 (87%) | 210 (87%) | 206 (85%) | 205 (85%) | 204 (85%) |
| Angiotensin receptor blocker | 202 (83%) | 202 (83%) | 198 (82%) | 200 (83%) | 200 (83%) |
| Beta-blocker | 11 (5%) | 10 (4%) | 11 (5%) | 12 (5%) | 11 (5%) |
| Hydrochlorothiazide | 41 (17%) | 40 (17%) | 38 (16%) | 38 (16%) | 40 (17%) |
| Patients grouped by medication changes, no. (%) |  | 9(3%) | 26 (11%) | 43 (18%) | 43 (18%) |
| Increase of dosage or agent | – | 6 (2%) | 14 (6%) | 24 (10%) | 22 (9%) |
| Decrease of dosage or agent | – | 3 (1%) | 12 (5%) | 19 (8%) | 21 (9%) |

Abbreviations: COVID-19, coronavirus disease 2019; GAD-7, generalized anxiety disorder scale-7.

Patients was classified into two groups as without anxiety (GAD-7 ≤4) and with anxiety (GAD-7 ≥5) on the basis of GAD-7 scale scores.

^*^ The pandemic timeline of the COVID-19 in China was classified as mentioned above.

**Supplementary Table 8. Patients attending online lectures in relation to average morning SBP during the COVID-19 period**

|  | **The average morning SBP (mm Hg), mean (95%CI)** | |  |
| --- | --- | --- | --- |
| **The COVID-19 period** | **Patients attending the online lectures** | **Patients not attending the online lectures** | ***P* value*** |
| Patients with anxiety (n=262) | 158 (60.3%) | 104 (39.7%) |  |
| Before the outbreak  (Oct 21^st^, 2019–Jan 20^th^, 2020) | 132.4 (131.0, 133.8) | 132.8 (131.1, 134.6) | 0.71 |
| During the outbreak  (Jan 21^st^– Mar 21^st^, 2020) | 133.3 (131.7, 135.0) | 132.3 (130.3, 134.3) | 0.42 |
| Patients without anxiety (n=3462) | 2132 (61.6%) | 1330 (38.4%) |  |
| Before the outbreak  (Oct 21^st^, 2019–Jan 20^th^, 2020) | 131.1 (130.7, 131.4) | 131.5 (131.0, 132.0) | 0.18 |
| During the outbreak  (Jan 21^st^– Mar 21^st^, 2020) | 131.2 (129.8, 130.5) | 130.6 (130.1, 131.1) | 0.19 |

Abbreviations: COVID-19, coronavirus disease 2019; SBP, systolic blood pressure; CI, confidence interval.

**P* value was calculated by linear regression model after adjustment for age, sex, and BMI.

**Supplementary Table 9. Change in physical activities in relation to average morning SBP during the COVID-19 period**

|  | **The average morning SBP (mm Hg), mean (95%CI)** | |  |
| --- | --- | --- | --- |
| **The COVID-19 period** | **Patients with decreased physical activities** | **Patients with unchanged physical activities** | ***P* value*** |
| Patients with anxiety (n=262) | 99 (37.8%) | 163 (62.2%) |  |
| Before the outbreak  (Oct 21^st^, 2019–Jan 20^th^, 2020) | 132.5 (130.7, 134.3) | 132.6 (131.2, 134.0) | 0.94 |
| During the outbreak  (Jan 21^st^– Mar 21^st^, 2020) | 133.3 (131.2, 135.3) | 132.7 (131.1, 134.3) | 0.67 |
| Patients without anxiety (n=3462) | 1446 (41.8%) | 2016 (58.2%) |  |
| Before the outbreak  (Oct 21^st^, 2019–Jan 20^th^, 2020) | 131.1 (130.7, 131.6) | 131.3 (130.9, 131.7) | 0.59 |
| During the outbreak  (Jan 21^st^– Mar 21^st^, 2020) | 130.2 (129.7, 130.7) | 130.4 (130.0, 130.8) | 0.48 |

Abbreviations: COVID-19, coronavirus disease 2019; SBP, systolic blood pressure; CI, confidence interval.

**P* value was calculated by linear regression model after adjustment for age, sex, and BMI.


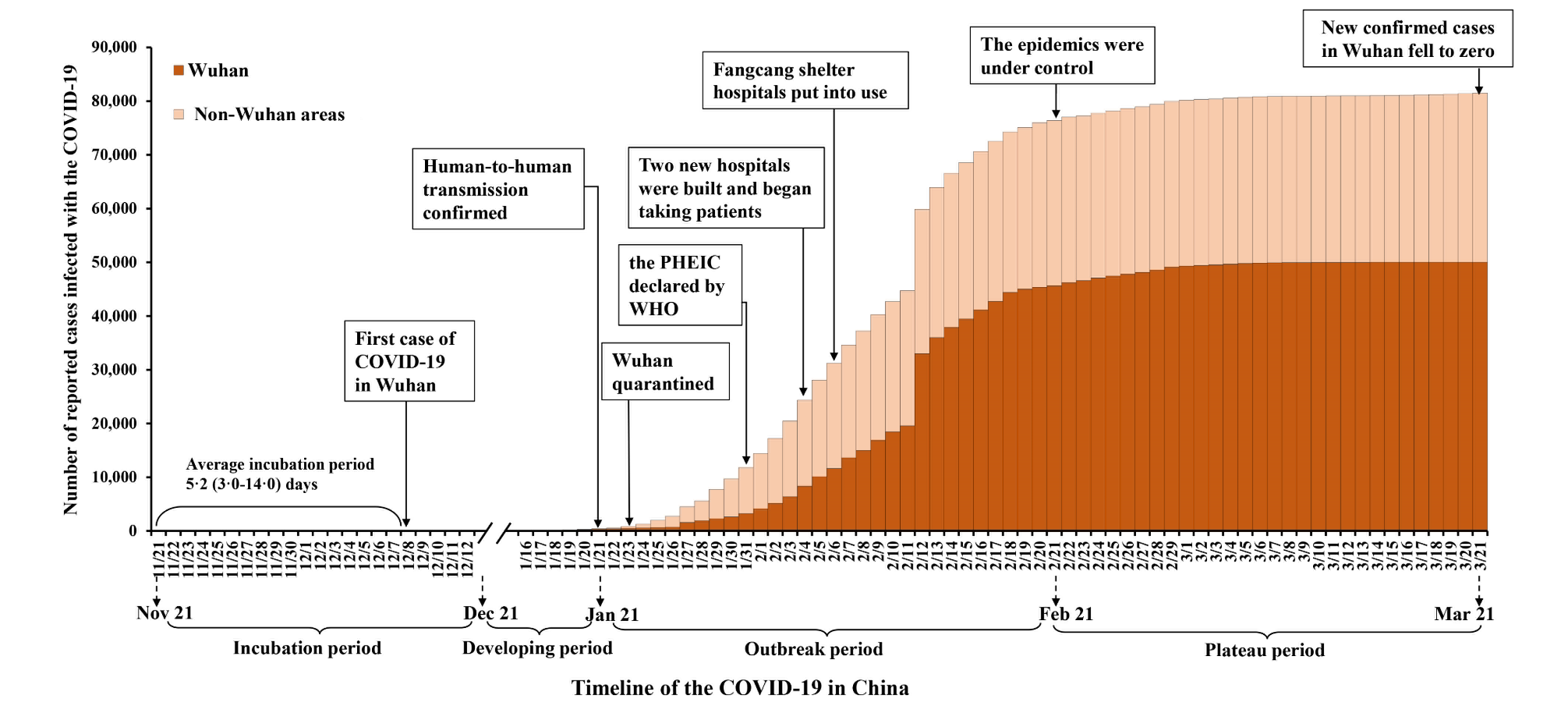


**Supplementary Figure 1. Timeline of the COVID-19 outbreak in Wuhan and other provinces of China**

Abbreviations: COVID-19, coronavirus disease 2019; WHO, World Health Organization; PHEIC, public health emergency of international concern.

**
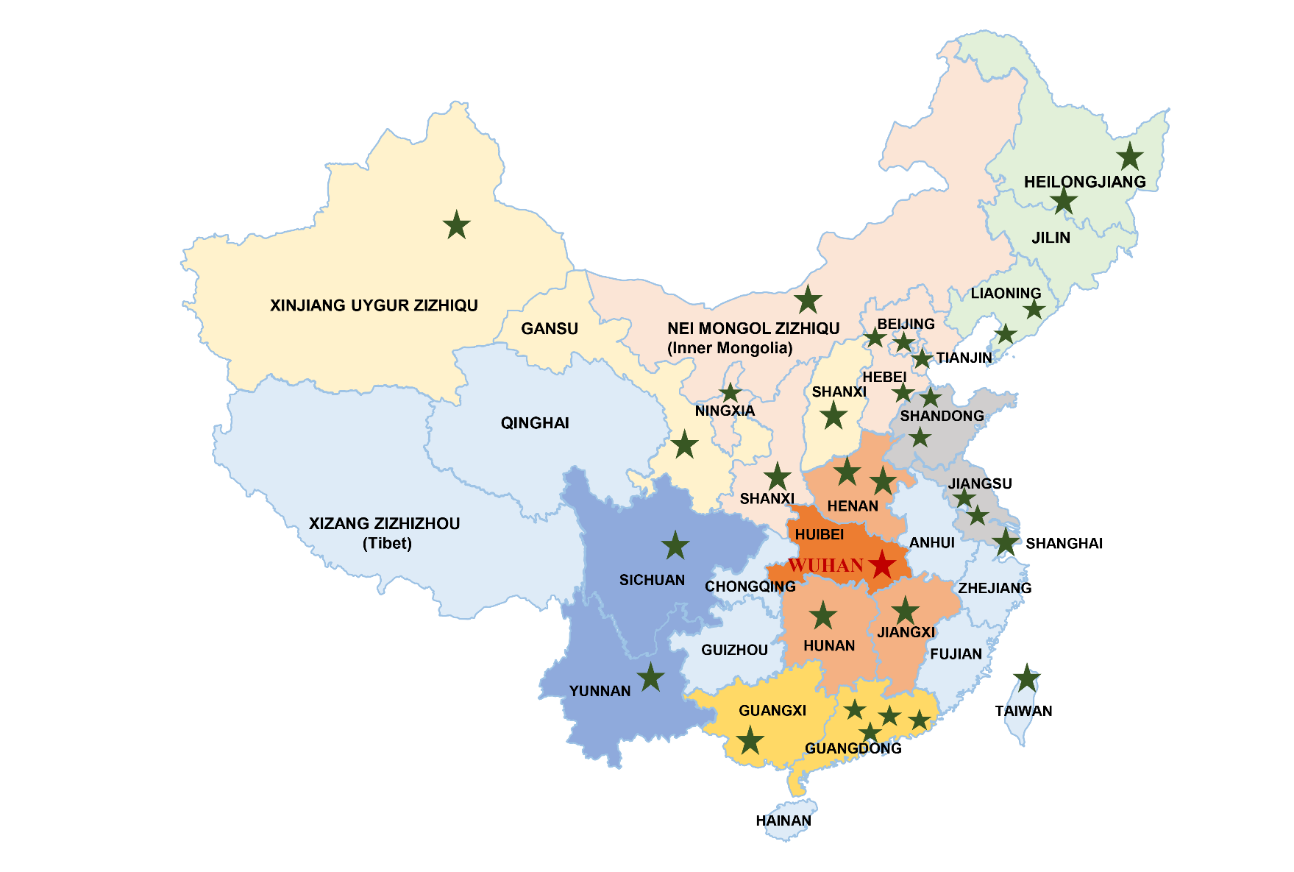
**

**Supplementary** **Figure 2. Geographical distribution of 42 participating hospitals in this study**


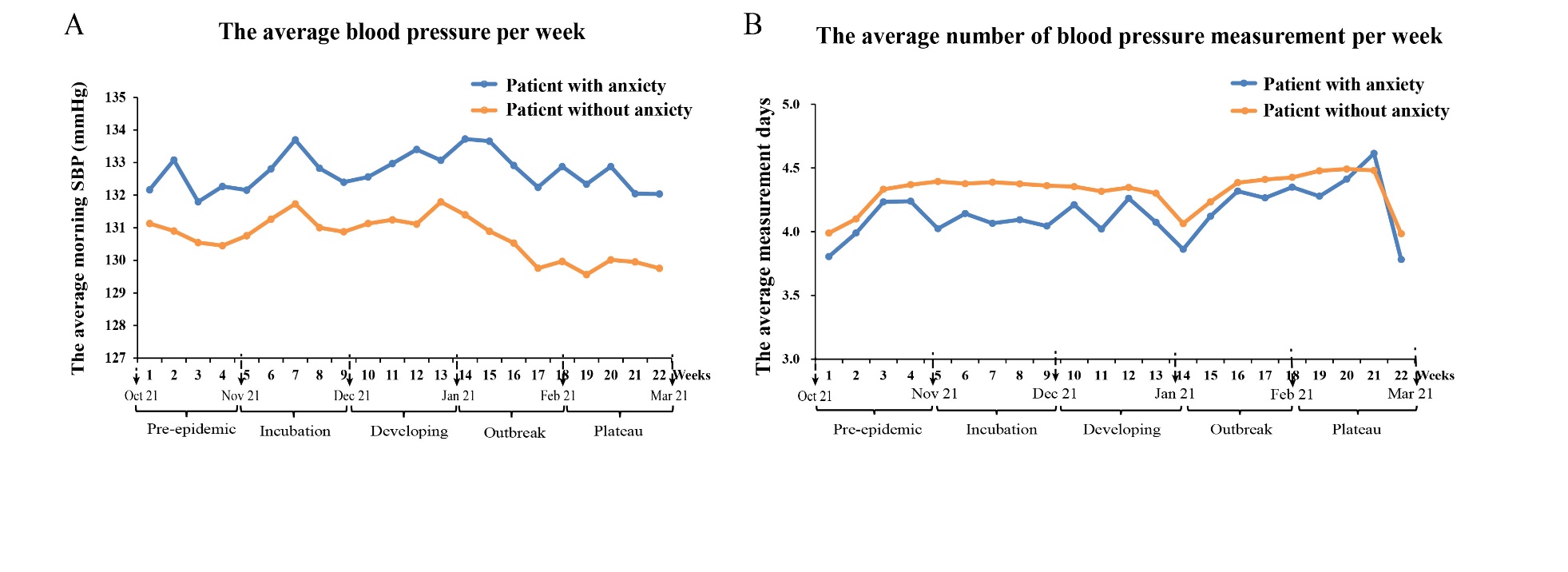


**Supplementary Figure 3. Average morning SBP and numbers of BP measurement per week in patients with anxiety and without anxiety during the COVID-19 pandemic**

Abbreviations: SBP, systolic blood pressure; COVID-19, coronavirus disease 2019.

A. The average morning SBP per week in patients with anxiety and patients without anxiety;

B. The average numbers of blood pressure measurements per week in patients with anxiety and patients without anxiety.


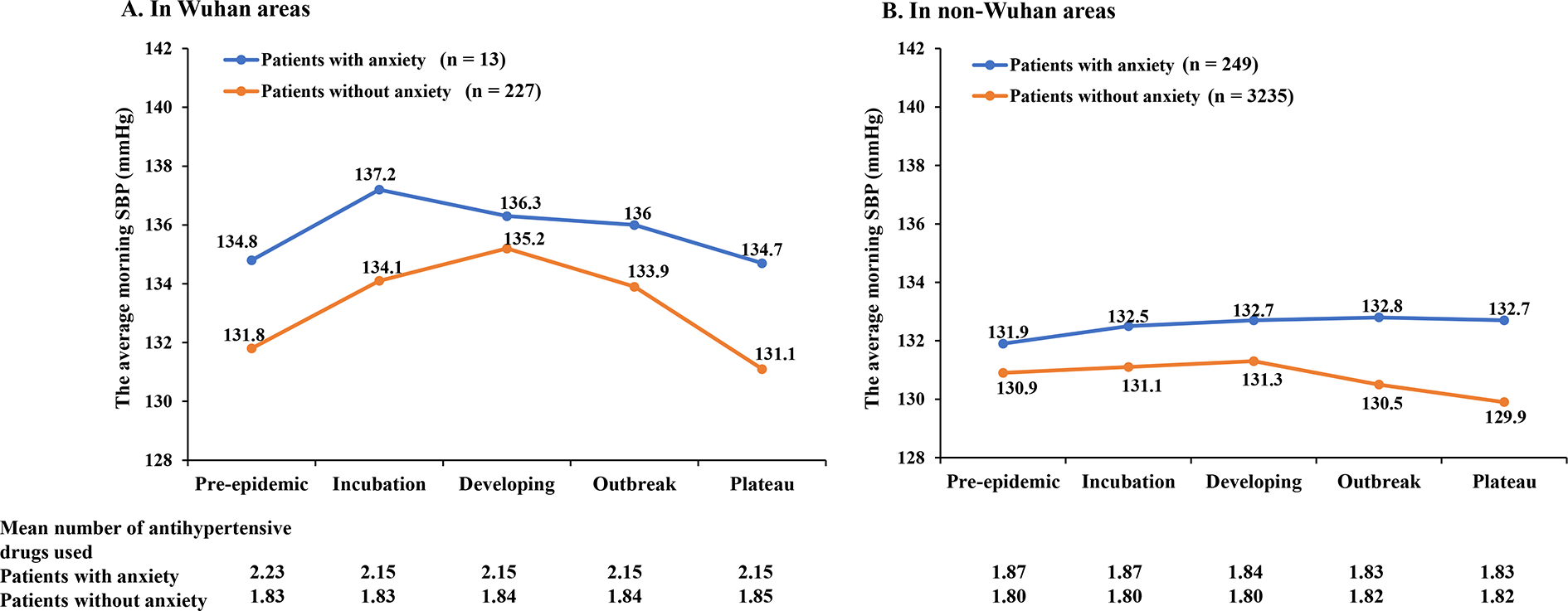


**Supplementary Figure 4. Trajectory pattern of morning SBP in patients with anxiety or without anxiety stratified by the Wuhan and non-Wuhan areas during the COVID-19 period**

Abbreviations: SBP, systolic blood pressure; COVID-19, coronavirus disease 2019.

The values in the graphs indicated as the adjusted mean of monthly morning SBP within each period of the pandemic among patients with anxiety (blue line) and without anxiety (orange line), after adjustment for age, sex, and body mass index.

A: The trajectory pattern of average morning SBP in Wuhan areas;

B. The trajectory pattern of average morning SBP in non-Wuhan areas.

**Supplementary**
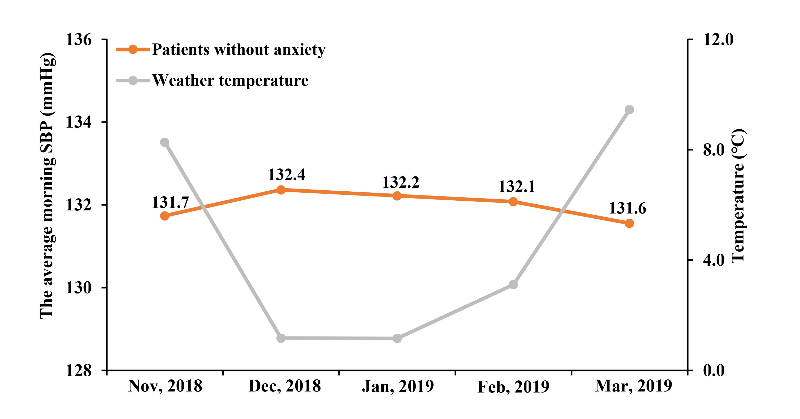
 **Figure 5. Seasonal variation in winter of average morning SBP in older patients in the previous year (November 20, 2018 to March 21, 2019)**

Abbreviations: SBP, systolic blood pressure.

The values in the graphs indicated as the adjusted mean of monthly average morning SBP among patients without anxiety (orange line). The grey line indicates the average temperature of the areas where the participants lived from November 2018 to March 2019.


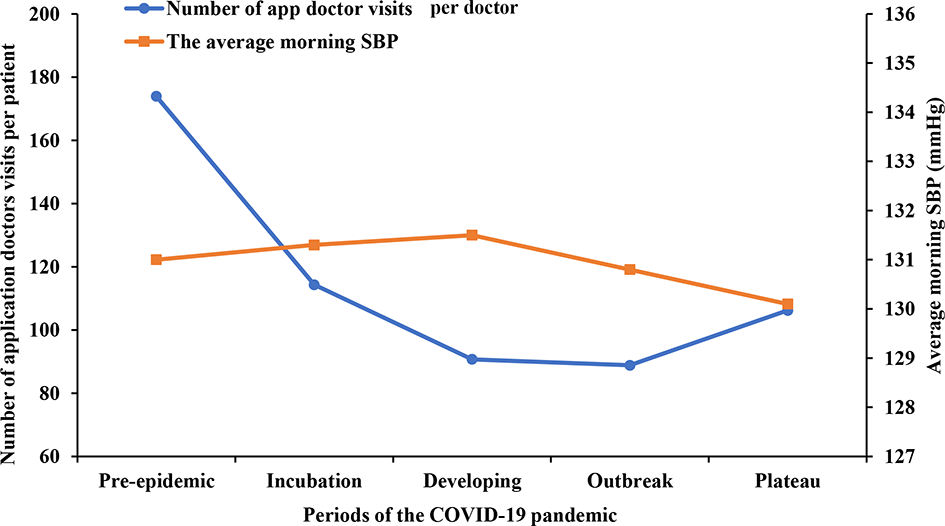


**Supplementary Figure 6. Fluctuations of average morning SBP in relation to the frequency of app visits by doctors during the COVID-19 period**

Abbreviations: SBP, systolic blood pressure; app, application; COVID-19, coronavirus disease 2019.


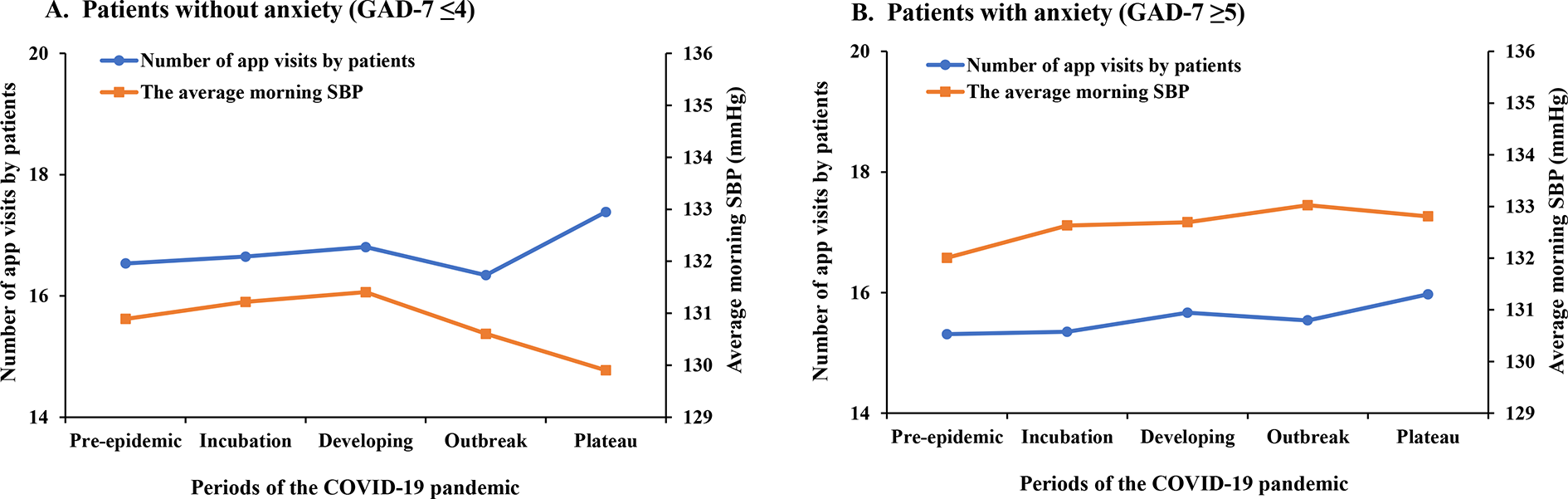


**Supplementary Figure 7. Fluctuations of average morning SBP in relation to the frequency of app visits by patients during the COVID -19 period**

Abbreviations: SBP, systolic blood pressure; app, application; COVID-19, coronavirus disease 2019; GAD-7, generalized anxiety disorder scale-7.

The frequency of checking blood pressure by patients through the app was higher for patients without anxiety (A) than patients with anxiety (B).

**Appendix 1. Questionnaire for health status of elderly patients with hypertension during the COVID-19 pandemic**
